# Design of trials for interrupting the transmission of endemic pathogens

**DOI:** 10.1186/s13063-016-1378-1

**Published:** 2016-06-06

**Authors:** Mariabeth Silkey, Tobias Homan, Nicolas Maire, Alexandra Hiscox, Richard Mukabana, Willem Takken, Thomas A. Smith

**Affiliations:** Swiss Tropical and Public Health Institute, Socinstrasse 57, Basel, CH-4002 Switzerland; University of Basel, Petersplatz 1, Basel, 4003 Switzerland; Wageningen University and Research Centre, Droevendaalsesteeg 4, Wageningen, 6708 Netherlands; ICIPE, Nairobi, PO Box 30772-00100 Kenya; University of Nairobi, Uhuru Highway, Nairobi, 00100 Kenya

**Keywords:** Stepped wedge design, Cluster randomization, Transmission model, Elimination, Vector control

## Abstract

**Background:**

Many interventions against infectious diseases have geographically diffuse effects. This leads to contamination between arms in cluster-randomized trials (CRTs). Pathogen elimination is the goal of many intervention programs against infectious agents, but contamination means that standard CRT designs and analyses do not provide inferences about the potential of interventions to interrupt pathogen transmission at maximum scale-up.

**Methods:**

A generic model of disease transmission was used to simulate infections in stepped wedge cluster-randomized trials (SWCRTs) of a transmission-reducing intervention, where the intervention has spatially diffuse effects. Simulations of such trials were then used to examine the potential of such designs for providing generalizable causal inferences about the impact of such interventions, including measurements of the contamination effects. The simulations were applied to the geography of Rusinga Island, Lake Victoria, Kenya, the site of the SolarMal trial on the use of odor-baited mosquito traps to eliminate *Plasmodium falciparum* malaria. These were used to compare variants in the proposed SWCRT designs for the SolarMal trial.

**Results:**

Measures of contamination effects were found that could be assessed in the simulated trials. Inspired by analyses of trials of insecticide-treated nets against malaria when applied to the geography of the SolarMal trial, these measures were found to be robust to different variants of SWCRT design. Analyses of the likely extent of contamination effects supported the choice of cluster size for the trial.

**Conclusions:**

The SWCRT is an appropriate design for trials that assess the feasibility of local elimination of a pathogen. The effects of incomplete coverage can be estimated by analyzing the extent of contamination between arms in such trials, and the estimates also support inferences about causality. The SolarMal example illustrates how generic transmission models incorporating spatial smoothing can be used to simulate such trials for a power calculation and optimization of cluster size and randomization strategies. The approach is applicable to a range of infectious diseases transmitted via environmental reservoirs or via arthropod vectors.

**Electronic supplementary material:**

The online version of this article (doi:10.1186/s13063-016-1378-1) contains supplementary material, which is available to authorized users.

## Background

Pathogen elimination is the goal of many intervention programs against infectious agents, such as mass chemotherapy, vaccine programs, behavioral change to reduce contacts, and vector control. The objective of interrupting transmission in whole populations impacts the choice of trial study designs. Typical before-and-after comparisons of populations have no replication and no contemporaneous control, and therefore, they have an effective sample size of one. If transmission continues post-intervention, it is impossible to know whether this was the result of bad luck. If transmission is successfully interrupted with a before-and-after design, it is unclear whether the intensity of intervention was appropriate or a massive overkill, or whether the disappearance of the pathogen was fortuitous. In such studies, it is not possible to distinguish changes in transmission due to the intervention from stochastic fluctuations in transmission levels or, when the pathogens are endemic, from environmental variation.

Randomization is critical if a study is to provide robust evidence of causality [1]. Where assignment at the individual level is impossible, cluster-randomized trials (CRTs) are often the best way to derive causal inferences about infrastructural or behavioral interventions. Clustering may be needed due to the nature of the intervention or where effects at the community level are anticipated that would be averaged across the whole population in an individual-level randomized trial [2–4]. CRTs are, therefore, the usual approach to achieve replication and contemporaneous controls in trials of infectious disease interventions, which typically provide both individual protection to the immediate recipients and also induce community effects by reducing onward transmission. Cluster size is critical in such trials: if the clusters are too small then the effect of the interventions will be propagated beyond the cluster edges via the community effect throughout the whole population. Such contamination effects bias the difference between the trial arms towards zero. If the clusters are too large, and hence few in number, there are insufficient degrees of freedom to distinguish the intervention effect from residual stochastic variation among clusters. Only with a sufficient number of adequately sized clusters is it possible to provide convincing inference.

Unfortunately, standard parallel CRT designs cannot provide a rigorous test of whether local elimination of a pathogen is feasible. This requires scale-up to universal coverage over the whole area, which cannot be achieved if there are untreated control clusters. For this purpose, we propose the stepped wedge cluster randomized trial (SWCRT), in which the intervention is introduced one cluster at a time until the whole area is covered. SWCRT elegantly combines the elements of group randomization, replication, contemporaneous controls, and complete coverage.

Population-based trials of infectious disease interventions do not directly estimate the efficacy of an intervention in reducing the rate of transmission that would be observed in a laboratory setting. This is both because interventions are generally not applied perfectly, and also because what is measured (the effectiveness) is generally the cumulative effects of recurrent transmission events, conditional on the pattern of contacts. Different effectiveness measures can be estimated for CRTs (and SWCRTs), either by comparing clusters before intervention with those that have already been intervened, or by comparing the whole study area with a non-intervention area (or possibly the same area, pre-intervention) [5, 6]. With an appropriate cluster size, the contamination effect can also be estimated from the range and gradient of the intervention effect across cluster boundaries. These possibilities are exemplified by an analysis of CRTs of insecticide-treated nets (ITNs) [7–9] for the control of malaria. These analyses confirm that if the central area of the intervention clusters is far enough away from the intervention boundaries, an estimate of the locally maximum intervention effect can be made, unaffected by contamination from control clusters. These studies also provide information about the effects of imperfect coverage, which can be used to parameterize process models for predicting the impact of sub-optimal deployment in other settings.

With SWCRT designs, while the individual cluster size may be approximately constant in terms of either area or population, the boundaries between the arms are constantly changing, and hence, the size of coalescing intervention areas increases during the study. Eventually, the entire population receives the intervention, so the maximal intervention population is obtained [10]. Thus, the overall size of such a trial is likely to be very large, with the costs of intervention deployment large in relation to those of data collection. An adequate sample size in terms of the total number of individuals enrolled or volume of data is a given [as in our application example, the Solar Power for Malaria Control trial (SolarMal)] and so these trials are likely to be powered to allow analysis of the temporal pattern of effectiveness. In this spirit, we evaluate designs under the assumption of one large overall sample size, as per section “Simulated trial designs for random geographies with uniform initial incidence”, so that the assessment of power is a comparison of power and time-dependent measures of effectiveness among designs rather than a calculation intended for estimating the absolute sample size needed to detect a given size of signal.

Empirical power and sample size calculations for CRT and SWCRT designs have been proposed [10, 11], but these do not directly address the issue of community effects, either in contaminating the control arm of the study or as a potential target for measurement. Our approach, to address the impact of community effects directly, is compatible with that of Halloran et al. [12], where each household in our simulation is a mini-community with its own population, and its own location relative to other mini-communities on the landscape.

In this paper, simulations of SWCRTs are used to consider how these designs might be analyzed to provide generalizable causal inferences about an intervention, giving particular consideration to the impact of variations in the cluster size relative to the extent of community effects. We use a generic model of disease transmission for the simulations, so that the results are broadly applicable to a range of infections transmitted either directly via environmental reservoirs or via arthropod vectors. Two new measures of effectiveness, inspired by analyses of CRTs of ITNs as protection against malaria infection, are proposed and their merits for inferring causality from the data produced in a SWCRT design are considered. The new measures are applied as an example to the design of a trial of odor-baited mosquito traps (OBTs) to reduce mosquito population size, reduce biting intensity, and eliminate *P. falciparum* malaria from Rusinga Island, Lake Victoria, Kenya (SolarMal) [13].

## Methods

### Simulation model of infection

The core of all simulations presented in this paper is a simple individually based susceptible–infected–susceptible model of infection transmission. The model does not aim to reproduce the within-host dynamics of any particular pathogen, since each infection is recorded only at one point in time, and each individual is available to be infected again at the next time step. The model aims to capture the force of infection at each time step before, during, and after the intervention is introduced across the study area. Once the behavior of the model is confirmed, then the theoretical impacts and interactions of the pathogen’s initial incidence, the extent of the community effect, and the efficacy of the proposed intervention are explored via simulations of three study design schemes for assigning sequences to clusters of uniform physical size. For this discrete time model, incidence is defined as the proportion of individuals with disease recorded at the specified time step. Empirical power estimates and confidence interval widths of model predictions are used to evaluate the proposed experimental designs, in terms of both optimal design structure and most informative measures of effectiveness. From these general results, a preferred design structure is selected for the SolarMal trial [13].

Discrete-time stochastic simulations of disease transmission are implemented using a one-week time step and a population of simulated individuals indexed with *i*, where *N*(*t*) is the cumulative number of individuals having received the intervention for the first time at time step *t*. The simulation approach is as follows:

- **1. Specification of simulated population:** A simulated population of total size *N*(*T*) is defined, where *T* is the last time step of the intervention. Simulated individuals are allocated to random point locations in a defined geometry.
- **2. Establishment of initial endemic stable state:** To initialize the simulation to a stable state, infections are independently assigned to each individual with a probability equal to a specified incidence, $\overline {y_{0}}$, for each week of the initial 10 weeks of the simulation.
- **3. Updating via transmission model:** For subsequent time points, *t*>10, new infections were generated via a two-state auto-regressive process with distributed lag, such that, for each individual *i* at time step *t*, the incidence is:
  1a $$ {\kern12pt} y(i,t) \sim \text{Bernoulli}\,(E[y(i,t)])  $$
  1b $$ E[y(i,t) ] = 1 - \exp{(- \beta_{0} \overline{y_{r}} (i, t))}  $$
  where *β*_0_, the transmission parameter, is the expected number of infectious contacts received by each host per time step. $\overline {y_{r}}(i,t)$ is the infectious reservoir for each simulated individual at time step *t*, defined as the percentage of infected members in its neighborhood:
  2 $$ \overline{y_{r}}(i,t) = { \sum\limits_{\tau = 6}^{10} w_{\tau} \frac{\sum\limits_{j} y(j,t-\tau)I_{r}(i,j)} { \sum\limits_{j} I_{r}(i,j) }}  $$
  where *I*_*r*_(*i*,*j*) is an indicator variable taking the value 1 if hosts *i* and *j* are located a distance less than *r* from each other, and is otherwise 0. The weights *w*_*τ*_ (which sum to 1) specify a kernel defining the lag times and vary between 6 and 10 time units (weeks). To align the transmission parameter with the predefined initial state and the constraint that transmission must be strictly positive, the parameter *β*_0_ is assigned a value based on the mean infectious reservoir across the whole study population at time 0, ${\overline {y_{r}}(0)}$:
  3 $$ \beta_{0} = -\frac { ln\,(1 - \overline{y_{r}}(0))} {\overline{y_{r}}(0) }  $$
  leading to a susceptible–infected–susceptible model of infection dynamics with the generation time distributed according to the lag.
- **4. Incorporation of intervention effects:** The direct effect of the intervention is to reduce the force of infection in the intervention clusters, by the protective efficacy against infection, so that the individual and time-specific transmission is modeled as:
  4a $$ {\kern12pt} y(i,t) \sim \text{Bernoulli}\,(E[y(i,t)])  $$
  4b $$ E[ y(i,t) ] = (1 - \exp{(- \beta_{0} \overline{y_{r}} (i, t) (1-C_{r} (i, t) E_{s}))}  $$
  where $\overline {y_{r}}(i,t)$ is defined as before and captures the state of the reservoir for each individual at each time step, *E*_*s*_ is the efficacy of the proposed intervention in protecting users from any single infection event (i.e., the proportionate reduction in the probability that infection occurs), and *C*_*r*_(*i*,*t*) is the percentage of each individual’s neighborhood that has received the intervention at time *t*. Thus, 1−*C*_*r*_(*i*,*t*)*E*_*s*_ represents the proportion of transmission that withstands the effect of the intervention.

#### Rationale

The generation time and spatial averaging of the infectious reservoir $\overline {y_{r}} (i, t)$ over each neighborhood is intended to approximate the spatial and temporal pattern of *P. falciparum* transmission. It is intended to approximate to proportionality the data that might be generated in a trial in which the outcome is the incidence of clinical disease, which in turn is assumed to vary proportionately to the force of infection. A latent period equivalent to six weekly time steps is simulated to capture the delay between the infection process and clinical disease and the approximate generation time of the infection. (This is a very simple approximation to the generation time of *P. falciparum* malaria.)

The simulation does not aim to capture the effects of the changing immune status during the trial, i.e., the transmission parameter *β* is held constant at *β*_0_. *E*_*s*_ can capture the effects achieved by reducing the infectious reservoir with chemotherapy, vaccines, and isolation of infectious cases, or by reducing the vectorial capacity for vector-borne diseases.

### Simulated trial designs for random geographies with uniform initial incidence

To evaluate the impact of various initialization parameters, ten island landscapes were simulated. For each landscape, 1000 households were allocated to random point locations in a square grid of dimension 9×9 km. Then, 4000 individuals were randomly assigned across these households, with each household constrained to have at least one member. Once the locations of these households were assigned, the neighbors of each inhabitant were calculated as all those individuals within a community of radius *r*, the maximum physical extent of the postulated community effect. The landscape description was completed by dividing the grid into 81 equal-area (but not equal-population) clusters and calculating a median location of all households within each cluster.

**Fig. 1:**
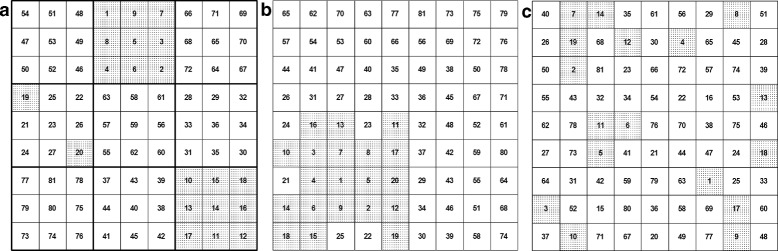
Schematics of three SWCRT design sequences on a grid of 81 equal-area clusters. Clusters are numbered in the order of the design rollout sequence. In the diagram as shaded, clusters 1–20 have received the intervention and clusters 21–81 have not yet received the intervention. All sequences begin at one randomly selected cluster. **a** Hierarchical SWCRT sequence: the sequence begins at one randomly selected meta-cluster. Clusters within that meta-cluster are filled in a random order until the meta-cluster is complete, then the next meta-cluster is selected. **b** Oil-drop SWCRT sequence: the sequence begins at one randomly selected cluster and spreads across adjacent clusters until the grid is filled. **c** Random SWCRT sequence: clusters are selected at random until the grid is completely filled. For all SWCRT designs, by the end of the intervention rollout there will be an equal number of cluster-days with and without the intervention. However, at almost all time points during the rollout, the populations in the two study arms will be unequal. *SWCRT* stepped wedge cluster randomized trial

Three possible CRT designs were simulated for each landscape: the *random*, the *oil drop*, and the *hierarchical* designs, represented schematically in Fig. 1. For the first design, the order in which clusters are selected to receive the intervention was completely random, i.e., the intervention sequences for the random design are single permutations of the cluster numbering. For the second design, a cluster was initially selected at random from among the 81 possible clusters; clusters were then chosen at increasing median cluster distance from the initial cluster, forming a single intervention zone that increased in size until the grid was completely covered. The third, hierarchical, design is a compromise between the random and the oil drop, motivated by the desire to retain comparators remote from the intervention while maintaining sufficient randomness not to bias experimental outcomes. For the hierarchical design, the grid was divided into nine equal-sized meta-clusters, which were further subdivided into nine equal-sized clusters. Hierarchical sequences were generated with the following algorithm: one cluster of the 81 was selected at random; all clusters within the same meta-cluster as the initial cluster were then selected at random until all had received the intervention and the meta-cluster was full. The next cluster was then chosen at random from the remaining 72 clusters. The procedure was repeated until all clusters in all meta-clusters on the grid had received the intervention. The relative randomness of these design structures can be stated in terms of the number of suitable sequences that could be generated for each design. A total of 81! possible sequences exist for the random design, 81 possible sequences exist for the oil-drop design, and (9)!×(9)! possible sequences exist for the hierarchical design.

The simulated intervention was introduced to all households within a single cluster during each time step. The duration of the introduction of the intervention across the study was 81 time steps (weeks), *T*=81. The total number of individuals in the study was *N*(*T*), so that at each time *t*, some number *n*(*t*) of individuals moved to the intervention arm, which had a total size at time *t* of $\sum \limits _{\tau =1}^{t} n(\tau)$. The non-intervention arm was divided into those who have neighbors in the intervention arm and were, thus, susceptible to first-order community effects and a pure comparator group who were neither recipients of the intervention nor neighbors of any recipients of the intervention. The total number of individuals in these three groups and the numbers of clinical cases occurring within each group (intervened, non-intervened but nearby, and non-intervened but remote) were tallied during the initialization period and at all subsequent time steps, as per Figure S2 in Additional file 1.

In total, 80 simulations were run for each of 100 randomly generated cluster allocation sequences corresponding to the random, oil drop, and hierarchical designs for 45 parameterizations comprising five levels of initial pathogen incidence (10 %, 20 %, 30 %, 50 %, and 80 %), three levels of neighborhood radius (0.5, 1.0, and 1.5 km), and three levels of intervention efficacy (0 %, 30 %, and 80 %) across ten randomly generated landscapes, where each landscape was a set of 1000 randomly distributed households across the island, with a total population of 4000 inhabitants.

### Simulated trials for non-uniform population densities and initial incidence

If there are underlying spatial trends in the disease, correlated with the spatial pattern of the rollout, it is difficult to interpret the results of a SWCRT. To evaluate the performance of the different designs in such situations, simulations were run assuming spatial heterogeneity in initial incidence, with a smooth spatial pattern in initial incidence described by bivariate probit distributions:

$$N \left(\mu_{1}, \mu_{2}, \left[\begin{array}{cc} 1 & \rho \\ \rho &1 \end{array}\right] \right) $$

and with the maximum incidence at a random location on the 9×9 km grid. These spatial distributions of infection were simulated with a range of different spatial patterns for the rollout of the intervention.

Similarly, heterogeneity in host population density might also affect the efficiency of different designs. To evaluate this, simulations were run assuming a population concentrated at the grid edges, a distribution, e.g., typical of many islands. Half of the households initially assigned to the 21 most central grid squares were reallocated to randomly sampled locations (and clusters) further from the center than this, thereby depleting the population in the core region. For these simulations, 700 randomization sequences corresponding to the three design structures were evaluated at one level of initial incidence (20 %), two levels of efficacy (30 % and 80 %), and one level of community radius (1 km) for a total population, *N*(*T*), of 4000 individuals. For each randomization sequence, 800 simulations were carried out.

### Intervention effectiveness measures

Following Halloran, Longini, and Struchiner [6, 14], a series of effectiveness measures, $\hat {e}_{1}(t)$, $\hat {e}_{2}(t)$, …, $\hat {e}_{6}(t)$, were computed from the results of the simulated trials. These include estimates of direct, indirect, and overall effects, and two novel measures, $\hat {e}_{5}(t)$ and $\hat {e}_{6}(t)$, that distinguish non-intervened individuals according to whether they are considered to be close to or remote from the intervention at time *t*. These measures, on which we propose to base inferences about intervention effects, are given in Table 1. To calculate these measures at each time step, the population was classified into categories for intervened, remote from the interventions, and neighboring the intervention but not yet intervened (see Figure S1 in Additional file 1). Three of the effectiveness measures, $\hat {e}_{1}(t)$, $\hat {e}_{2}(t)$, and $\hat {e}_{3}(t)$, involve comparisons with the baseline mean outcome at each time step, which is the incidence at the time step before the first introduction of the intervention to the island computed as

**Table 1:** Intervention effectiveness measures, as adapted from [14]

Measure	Intervention	Mean outcome	Comparator group	Time-dependent effectiveness measure
number	group	intervention group		
Baseline comparison groups				
1	Intervened	$ \frac { \sum _{i} y(i,t)I(i,t)} {N(t)} $	Baseline	$ \hat {e}_{1}(t) ~=~ 1 - \frac { \sum _{i} y(i,t) I(i,t)} { \overline {Y}_{b} N(T)} $
2	Naive	$ \frac { \sum _{i} y(i,t)(1-I(i,t))} {N(T)- N(t)} $	Baseline	$ \hat {e}_{2}(t)~=~ 1 - \frac { \sum _{i} y(i,t)(1-I(i,t))} {\overline {Y}_{b}(N(T)- N(t))} $
3	Trial population	$ \frac { \sum _{i} y(i,t)} {N(T)} $	Baseline	$ \hat {e}_{3}(t) ~=~ 1 - \frac { \sum _{i} y(i,t)} { \overline {Y}_{b} N(T)} $
Contemporaneous comparison groups				
4	Intervened	$\frac { \sum _{i} y(i,t)I(i,t)} {N(t)}$	Naive	$ \hat {e}_{4}(t)~=~1 - \frac {(N(T)- N(t)) \sum _{i} y(i,t)I(i,t)} {N(t) \sum _{i} y(i,t) (1-I(i,t)) }$
5	Intervened	$ \frac { \sum _{i} y(i,t)I(i,t)} {N(t)} $	Naive remote from intervention	$ \hat {e}_{5}(t) ~= ~1 - \frac {\sum _{i}y(i,t)I(i,t) \sum _{i} (1-I^{*}(i,t))} { N(t) \sum _{i} y(i,t)(1-I^{*}(i,t))} $
6	Naive close to	$ \frac { \sum _{i} y(i,t)(1-I(i,t))I^{*}(i,t)} { \sum _{i} (1-I(i,t))I^{*}(i,t)} $	Naive remote from intervention	$ \hat {e}_{5}(t) ~= ~1 - \frac {\sum _{i} y(i,t)(1-I(i,t))I^{*}(i,t) \sum _{i} (1-I^{*}(i,t))} {\sum _{i} y(i,t)(1-I(i,t))I^{*}(i,t) \sum _{i} y(i,t)(1-I^{*}(i,t))} $
	intervention			

*y*(*i*,*t*): Outcome measured for individual *i* at time *t*. $\overline {Y}_{b}$: Mean outcome at baseline. *N*(*t*): Total number of individuals in intervened clusters at time *t*. *I*(*i*,*t*): Indicator taking value 1 if individual *i* is in an intervened cluster at time *t*, 0 otherwise. *I* ^∗^(*i*,*t*): indicator taking value 1 if individual *i* is intervened or less than distance *r* from the nearest intervened cluster at time *t*, 0 otherwise, so that (1−*I*(*i*,*t*))*I* ^∗^(*i*,*t*) indicates those individuals in the naive close to intervention category Following Halloran [6], effectiveness measures $\hat {e}_{1}$, $\hat {e}_{2}$, and $\hat {e}_{4}$ are direct measurements of intervention effectiveness, of which only $\hat {e}_{4}$ is contemporaneous. $\hat {e}_{3}$ is the overall measure of effectiveness for a before-and-after study. $\hat {e}_{5} $ and $\hat {e}_{6} $ are novel contemporaneous measurements that separate the direct and indirect effects during intervention rollout, avoiding the bias caused by contamination of the comparator group

5 $$ \overline{Y}_{b}= \frac { \sum\limits_{\tau=-b}^{\tau=-1} \sum\limits_{i} y (i,\tau)} {bN(T)}  $$

where *b* is the number of time steps included in the baseline, *y*(*i*,*t*) is the observed value of the outcome (i.e., presenting with the disease or not), and *N*(*T*) is the total population at risk.

$\hat {e}_{4}(t)$, $\hat {e}_{5}(t)$, and $\hat {e}_{6}(t)$ are contemporaneous measures of effect that depend on the randomized assignments of clusters, and so are particularly relevant for causal inference. The standard contemporaneous direct effectiveness measure, $\hat {e}_{4}(t)$, directly compares the clinical case rate in the intervened and non-intervened populations at each time step. We propose a new direct effectiveness measure, $\hat {e}_{5}(t)$, shown in Table 1, which restricts the contemporaneous comparator group to those hosts located remotely from the intervention. While $\hat {e}_{5}(t)$ estimates the direct effect of the intervention, as the trial proceeds this becomes the cumulated effect of many transmission events (so it is not an estimate of the efficacy *E*_*s*_ used in the generation of the simulated trials). We also define a new indirect effectiveness measure, $\hat {e}_{6}(t)$, applying the same contemporaneous comparator group as $\hat {e}_{5}(t)$ to measure the influence of the intervention in the non-intervened group (i.e., the community effect). As before, *remote* is strictly defined as all members of the non-intervened group who have no neighbors in the opposite arm of the trial at a given time step, where neighbor status is determined from the given community radius, *r*, beyond which the spillover effect of the intervention is anticipated to be negligible. In practice, the community radius must be defined on the basis of observations from previous trials or the biology of the pathogen. Randomness in the infection process cannot be separated from sampling variation. To enable comparisons among effectiveness measures for these simulations, the population at risk was equivalent to the total simulated population, *N*(*T*), fixed at a value of 4000, and the data from all simulated individuals contributed to the effectiveness calculations. We further considered a range of *r* values, where community membership for each individual is defined at each time step. *I*^∗^(*i*,*t*) is an indicator, taking the value 1 if *x*(*i*,*t*)≤*r* and 0 if *x*(*i*,*t*)>*r*.

Each of these six time-specific effectiveness estimates, evaluated at each time step during the simulation, is of the form:

$$\hat{e}(t) = 1 - \frac { Y_{1}(t)} { Y_{0}(t)} $$

where *Y*_0_(*t*) and *Y*_1_(*t*) are risks or rates in the comparator and intervention groups, respectively. Corresponding to each of these measures, cumulative effectiveness measures can be computed as:

$$\hat{E}(t) = 1 - \frac{ \sum\limits_{\tau=0}^{\tau=t} { Y_{1}(\tau) }} { \sum\limits_{\tau=0}^{\tau=t} { Y_{0}(\tau)} } $$

where both the numerator and denominator are summed over all time points up to *t*. An overall value for each measure is obtained by cumulating up to the end of the trial.

### Confidence intervals

In a real trial, $ \sum \limits _{\tau =0}^{\tau =t} { Y_{1}(\tau) }$ and $\sum \limits _{\tau =0}^{\tau =t} { Y_{0}(\tau) }$ are estimated from proportions of tested individuals positive for the infection or disease. Estimates of the ratio of these two proportions, and hence of the cumulated or overall effectiveness,

$$\hat{E}(t) = 1 - \frac{ \sum\limits_{\tau=0}^{\tau=t} { Y_{1}(\tau) }} { \sum\limits_{\tau=0}^{\tau=t} { Y_{0}(\tau)} } $$

(see above), can thus be made using logistic regression models, with random effect terms to allow for temporal variation, cluster differences in incidence, and if necessary for re-testing of the same individuals at repeated time points. Approximate model-based confidence intervals for the ratio of the two proportions and, hence, for the effectiveness, can then be made using the delta method [15].

To compare simulated trials, the distribution of effectiveness measures and their confidence intervals were calculated by carrying out 1000 independent simulations of each trial and analyzing the empirical distributions of the outcomes.

### Power, design, and sequence evaluation

A characteristic of the SWCRT design is that, as the membership of the populations shifts from non-intervened to intervened at each time step, so does the power of the chosen effectiveness measure. Point estimates were made from the simulations for each of the six effectiveness measures, and the power of each design was estimated for each time step. In each case, the same radius, *r*, was used for defining neighbors in the calculation of effectiveness measures $\hat {e}_{5}(t)$ and $\hat {e}_{6}(t)$ as was used in generating the simulations (in an actual field trial, the effects of using different radii to define neighbors will be analyzed to estimate the best fitting *r*). Empirical two-sided 90 % confidence intervals of direct comparisons with baseline $\hat {e}_{1}(t)$ and $\hat {e}_{2}(t)$, and indirect comparisons with baseline $\hat {e}_{3}(t)$ and the contemporaneous $\hat {e}_{4}(t)$, $\hat {e}_{5}(t)$, and $\hat {e}_{6}(t)$ effectiveness measures were drawn at each time step across all simulations. Results for the randomly generated sequences corresponding to the three different types of designs are ranked inversely by confidence interval half-width.

We derived the power estimates by comparing simulation results run under the null (*H*_0_: *E*_*s*_=0) and two alternative hypotheses (*H*_*a*_: *E*_*s*_=0.30 and *H*_*a*_: *E*_*s*_=0.80). Specifically, for each effectiveness measure, the 95 % quantile of the empirical null distribution was taken as an estimate of the critical value corresponding to a type I error of 5 % (*α*=5*%*) for a one-sided test. This value directly corresponds to the critical value under the alternative hypothesis. The value of *β* for each effectiveness measure (both summarized over the whole trial duration and specific to a time step) was calculated as the area-under-the-curve to the left of the critical value under the alternative (empirical) distribution. The power for each effectiveness measure at each time step was then calculated as 1−*β*.

All simulations were carried out at the High Performance Computing Core at the University of Basel in R version 3.02. Each simulation required only a few minutes of processing time.

### Simulated trial design for the SolarMal trial

A baseline health and demographic surveillance survey was carried out from May to July 2012 on Rusinga Island, which enumerated 4062 households with a total membership of 23,337 inhabitants. Approximately 22 % of the residents were diagnosed via rapid diagnostic tests as infected with *P. falciparum*.

The cluster size for the trial was matched to the logistical limit of the number of households that could receive the intervention within a week (i.e., 50). Thus, in contrast to the simulations of regular grids, in the application, the clusters were of approximately equal population but not equal geographic size. A minimal spanning tree algorithm [16], used to solve the classical traveling salesman problem, provided an optimal one-way path among households across Rusinga. The 4062 households along the path defined by the minimal spanning tree were then counted off along the path into 81 clusters, 12 of which were randomly selected to be assigned a total of 51 households, the remainder having 50 households assigned. A large number of randomizations, each consisting of an ordering of the 81 clusters thus defined, were randomly generated, corresponding to either hierarchical, oil-drop, or random SWCRT designs. For the hierarchical designs, contiguous sets of nine clusters were amalgamated into single meta-clusters (see Figure S2 in Additional file 1). A trial, involving the rollout of one cluster per week and based on each randomization, was simulated, with each of the 23,337 individuals on the island modeled as a single stochastic element. At each time step, individuals were identified within one of three groups: intervened, non-intervened but within the community radius of at least one intervened individual, or non-intervened beyond the community radius of any intervened individual, and each of the effectiveness measures listed in Table 1 was computed.

To classify individuals into these groups, pairwise great-circle distances among all households were calculated, and used as a basis for identifying all the neighbors within the community radius, *r*, for each individual within each household. A value of 1 km for *r* was used, based on the approximate scale of the effects in the trials of ITNs [7, 8]. The percentage of infections, *I*_*r*_(*i*,*j*), averaged across an individual’s neighborhood, was fed into the calculation for the infective reservoir at each time step. Likewise, intervention coverage rates, *C*_*r*_(*i*,*t*), for the neighborhood of each house were calculated for each time step and fed into the effectiveness calculation (Eq. 4a).

### Analysis of effectiveness for the simulated trial design for SolarMal and sequence selection

Point estimates and empirical 95 % confidence intervals of the six direct and indirect effectiveness measures were drawn for each time step from a set of 1000 independent replications of the simulated trial. The duration of utility of a given effectiveness measure is also of interest and is defined as the number of weeks from the start of introduction of the OBTs until the confidence interval width of an effectiveness measure increased to 10 %. A further ranking was made in order of total area under the confidence interval width versus time step curve from the 18th to the 65th week of the 81-week rollout (complete coverage). For this ranking procedure, confidence interval widths from the first and last 2 months were discarded as either the treatment or comparison groups were tending to zero and the effectiveness measures began to fluctuate wildly. Among those sequences with good statistical properties, additional sociological constraints were applied to select a group of sequences acceptable to a community stakeholder council; in particular, the intervention schedule should be constrained so that entire villages receive the intervention within 6 months.

## Results

### Model confirmation and explanation of the interrelationship between effectiveness measures

**Fig. 2:**
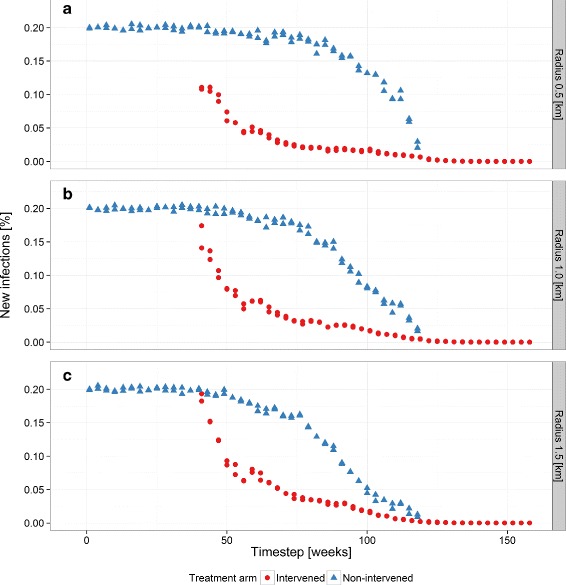
Example single random SWCRT sequence runs of the transmission simulator. Incidence of clinical events in intervened (*red*) and non-intervened (*blue*) populations as modeled by the transmission simulator for three levels of community radius, **a** 0.5 km, **b** 1.0 km, and **c** 1.5 km. The cluster width is held constant at 1 km, corresponding to an area of 1 km^2^. The transmission model input efficacy is 80 %. During the first 40 time steps of each simulation, the incidence of clinical events is an auto-regressive moving average process that oscillates around the initial incidence value of 20 %. The intervention commences at time step 41 and from time steps 41 to 121, the incidence of the pathogen decreases sharply in both arms due to the direct effect of the intervention and the community effect. The community effect has more impact at greater radii. *SWCRT* stepped wedge cluster randomized trial

Comparison of the average incidence in the intervention arm with that in the non-intervention arm in illustrative simulations (Fig. 2) clearly indicates that the transmission simulator can capture the main features that we would expect of a trial that succeeds in interrupting, or nearly interrupting, transmission of a pathogen. There was considerable variation in the incidence in the control arm in the first part of the intervention period (following time step 40). Only a very small number of individuals were initially included in the intervention arm. The decrease in incidence in the intervention arm was then rapid, and only after about a further 20 time points was an effect on the non-intervention arm evident. As the intervention was rolled out further, the infection was almost eliminated from the intervention arm, while the incidence in the control arm became highly variable between time points, presumably as a result of the reduced sample size in this arm. Incidence in the intervention arm continued to decrease, even once 100 % coverage was achieved, eventually reaching zero. This reflected the delay in the system resulting from the assumed generation time of the infection, together with the fact that the final extinction event was stochastic.

**Fig. 3:**
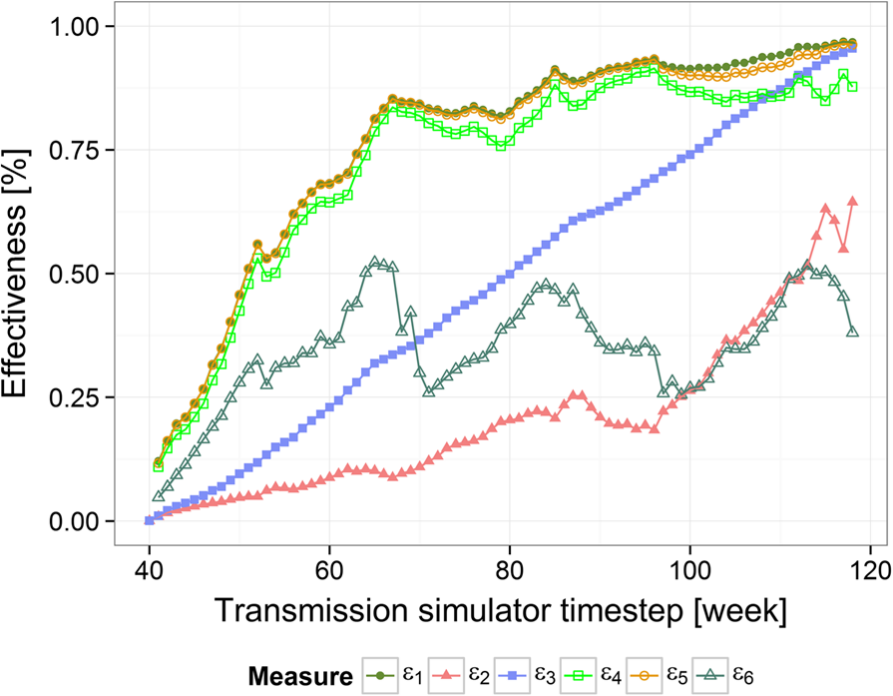
Relationships for the six effectiveness measures from Table 1 during a single random SWCRT sequence run of the transmission simulator. $ \hat {e}_{1}(t) $ (*filled green circles*) is a direct comparison between outcomes in the intervened group versus the status at baseline, $ \hat {e}_{2}(t) $ (*filled pink triangles*) is a direct comparison between outcomes in the non-intervened group versus the status at baseline, and $ \hat {e}_{3}(t) $ (*filled blue squares*) is an overall comparison of the entire study area versus baseline. $ \hat {e}_{4}(t)$ (*bright green squares*) is a direct comparison between the intervened and all non-intervened, $ \hat {e}_{5}(t)$ (*gold circles*) is a direct comparison between the intervened and those remote from the intervention, and $ \hat {e}_{6}(t)$ (*dark green triangles*) is a direct comparison between non-intervened populations close to and remote from the intervention. *SWCRT* stepped wedge cluster randomized trial

The effectiveness measures, computed for a specific time from a single theoretical random design simulation in which $\overline {y}(0) = 0.2$, *E*_*s*_=0.8, and *r*=1 km, are shown in Fig. 3. During the initial ten time steps after the introduction of the intervention, the direct effectiveness measures $\hat {e}_{1}(t)$, $ \hat {e}_{4}(t)$, and $ \hat {e}_{5}(t)$ were much lower than the efficacy in preventing infection since many of the infections at the start of the implementation were received before the hosts joined the intervention arm. These infections were initially pre-patent, that is, pre-symptomatic. Once the pre-patent period was exceeded, the direct effectiveness estimates rapidly reached and then exceeded the efficacy against infection, reflecting the cumulative effect on multiple generations of parasites.

The indirect baseline $\hat {e}_{2}(t)$ and direct contemporaneous $ \hat {e}_{4}(t)$ effectiveness measures diverged quickly at the beginning of the simulation and converged at the end of the simulation run. The baseline measure $ \hat {e}_{2}(t)$ was initially much lower than the direct effectiveness, and first climbed steeply towards the end of the simulation, when most residual non-intervention areas were close to the intervened clusters. Reflecting that the non-intervention arm was only a small part of the simulation at the start (when there was a low indirect effect) but predominated at the end (when there were few infections to avert), the addition of the indirect effect into the effectiveness calculation made little difference, so that when direct and indirect effects (computed by comparison with the baseline) were added together, the effectiveness profile was similar to that for the direct effect alone. The proposed contemporaneous indirect effectiveness measure $ \hat {e}_{6}(t)$ initially climbed quickly (within the first 2 months of rollout) to its maximum value and then oscillated due to sample size fluctuations as new clusters were brought into the intervention arm.

The overall effect $ \hat {e}_{3}(t)$, computed by comparison with the baseline, was dominated by the effect of the scale-up of the intervention and therefore, increased approximately linearly with time.

Cumulation of the numerators and denominators of the effectiveness estimates led to smoother curves than those in Fig. 3, each of them tending towards a clear value at the end of the intervention. Cumulation did not change the inferences to be made by examining each measure independently.

### Results from design and landscape simulations

In the simulated trials, regardless of initial parameterization or landscape (uniform or random hotspot, random geography, or central depletion geography), the simulated interventions in all cases had a cumulative impact of eliminating the pathogen by the end of the rollout. Details of the effectiveness measures and power computed from the simulations are given in the tables provided in Additional file 1. In all cases, the efficacy estimates and power of comparisons against baseline measures is high because the sample size of the comparator group is the largest possible—i.e., the entire study population.

**Fig. 4:**
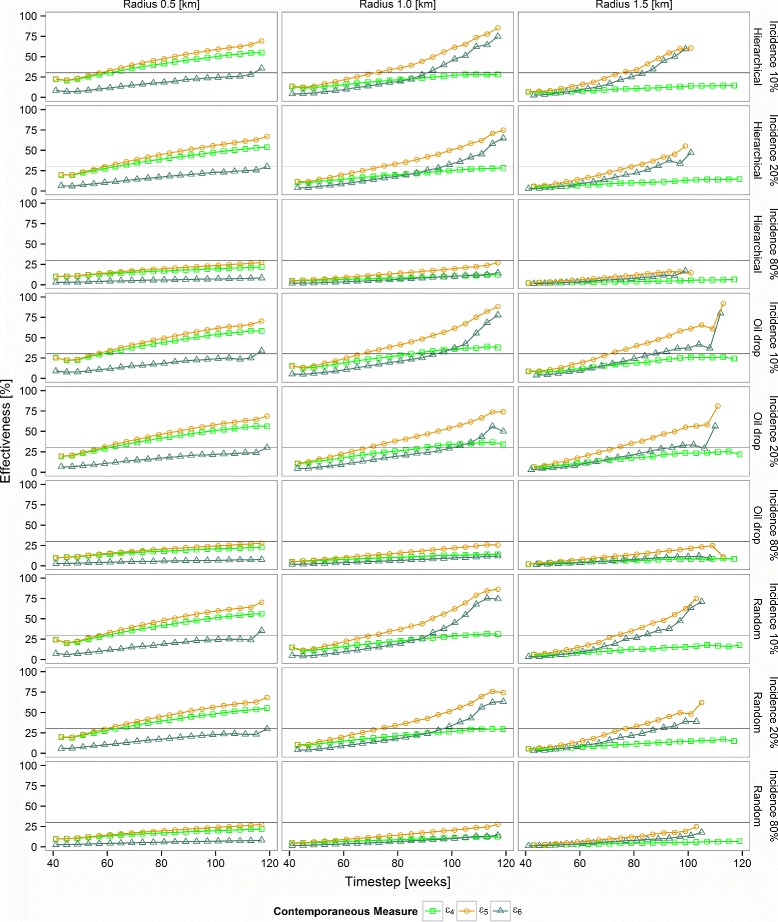
The three contemporaneous effectiveness measures over time. *ε*_4_ (*bright green squares*), *ε*_5_ (*gold circles*), and *ε*_6_ (*dark green triangles*). The *horizontal lines* correspond to the simulated efficacy *E*_*s*_=30 %

For all design structures and radii of effect, *r*, the values of initial incidence, *ε*_5_, are higher than the other measures of contemporaneous effectiveness (i.e., the gold line is always above the light green and dark green lines in Fig. 4). This is because *ε*_5_ compares intervened individuals with only those naive individuals remote from any contamination effects, and for whom, therefore, the intervention effects are minimal. In contrast, the comparator group for *ε*_4_, the conventional CRT effectiveness measure, contains individuals influenced by the spatial effect of the intervention and so measures an effect diluted by contamination. *ε*_6_ measures the magnitude of this contamination effect, and so increases when *ε*_4_ and *ε*_5_ diverge. Similar trajectories of these measures over time were observed for each of the three designs, but the effectiveness increased much more steeply over time when the initial incidence was low, and increased only gradually with $\overline {y_{0}} = 80$ %.

The optimal cluster size is one in which the direct and contamination effects are clearly separable, so an appropriate cluster size achieves high values of *ε*_6_ and large differences between *ε*_4_ and *ε*_5_. In our simulations, this corresponds most closely to clusters of width equal to the radius of the contamination, *r*. With clusters larger than this (i.e., the analyses with *r*=0.5 km, equivalent to half the cluster width), *ε*_6_ remains low, because there is relatively little contamination effect. With small clusters relative to the radius (i.e., the analyses with *r*=1.5 km), the estimated direct effect of the intervention *ε*_4_, corresponding to the conventional result, is much lower than *ε*_5_ in most of the simulations (Fig. 4), because the effect of the intervention spreads out across the whole surface.

Particular interest lies in the statistical power of the contemporaneous comparisons during the rollout, where the results are not easy to predict heuristically, because the relative power of the measures is constantly varying. Analyses considering a single time point at time step 60 (Table 2), indicate that among the contemporaneous measures, the one employing the remote comparator, $ \hat {e}_{5}(t) $, is generally of higher power than the direct comparison of intervened and non-intervened naive clusters $ \hat {e}_{4}(t) $ (Table 2). At *r* equal to the cluster width, $ \hat {e}_{4}(t) $ is the most powerful outcome, followed by $ \hat {e}_{5}(t) $, then $ \hat {e}_{6}(t) $. At higher *r* (corresponding to a greater degree of spatial smoothing of the intervention effects), $ \hat {e}_{4}(t) $ generally has lowest power. In general, power decreases with increasing baseline incidence, $\overline {y_{r}}(0)$, and correlates positively with intervention efficacy. While the power of outcome $ \hat {e}_{6}(t) $ does not show a clear relationship with the design type, the power of $ \hat {e}_{4}(t) $ and $ \hat {e}_{5}(t) $ is generally somewhat higher with the oil-drop design, followed by the hierarchical, and then the random order, though the differences are small.

**Table 2:** Power of three contemporaneous effectiveness measures at week 60, midway through the intervention rollout, type I error =10 % and efficacy *E*_*s*_=30 %

*r*	$\overline {y_{r}}(0)$	Power $\hat {e}_{4}$	Power $\hat {e}_{5} $	Power $\hat {e}_{6}$
Hierarchical design				
0.50	0.10	0.99	0.90	0.51
	0.20	0.99	0.90	0.52
	0.50	0.98	0.83	0.41
	0.80	0.85	0.59	0.18
1.00	0.10	0.83	0.82	0.67
	0.20	0.87	0.74	0.56
	0.50	0.81	0.64	0.45
	0.80	0.53	0.45	0.29
1.50	0.10	0.58	0.95	0.91
	0.20	0.59	0.86	0.78
	0.50	0.50	0.62	0.51
	0.80	0.28	0.45	0.37
Oil-drop design				
0.50	0.10	0.99	0.93	0.53
	0.20	0.99	0.94	0.54
	0.50	0.98	0.88	0.41
	0.80	0.86	0.64	0.18
1.00	0.10	0.91	0.84	0.64
	0.20	0.93	0.82	0.59
	0.50	0.88	0.74	0.48
	0.80	0.60	0.52	0.28
1.50	0.10	0.75	0.84	0.63
	0.20	0.77	0.82	0.61
	0.50	0.69	0.76	0.51
	0.80	0.41	0.52	0.31
Random cluster design				
0.50	0.10	0.98	0.89	0.52
	0.20	0.99	0.90	0.52
	0.50	0.97	0.83	0.40
	0.80	0.84	0.58	0.18
1.00	0.10	0.80	0.80	0.66
	0.20	0.84	0.73	0.58
	0.50	0.79	0.63	0.45
	0.80	0.50	0.44	0.29
1.50	0.10	0.54	0.94	0.88
	0.20	0.54	0.79	0.74
	0.50	0.45	0.56	0.48
	0.80	0.25	0.41	0.36

**Fig. 5:**
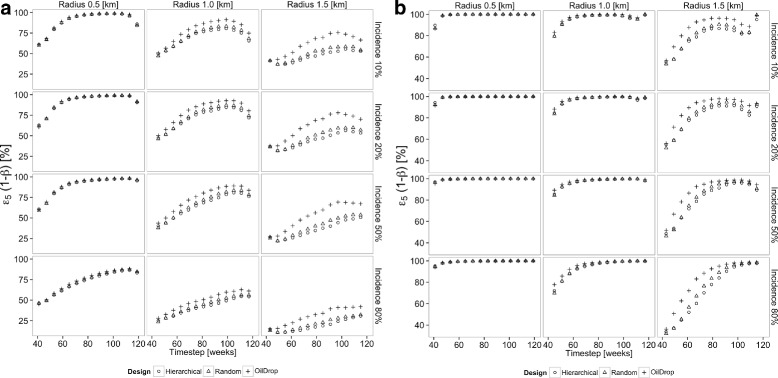
Power over time of contemporaneous effectiveness measure *ε*_5_(*t*) to detect a difference between the intervened treatment arm and non-intervened arm remote from the intervention. All simulations are based on a cluster diameter of 1 km and one-sided type I error rate *α*=5 %. Open circle: hierarchical ordering; plus sign: oil-drop ordering; open triangle: random ordering. **a** Intervention efficacy of 30 %; **b** intervention efficacy of 80 %

**Fig. 6:**
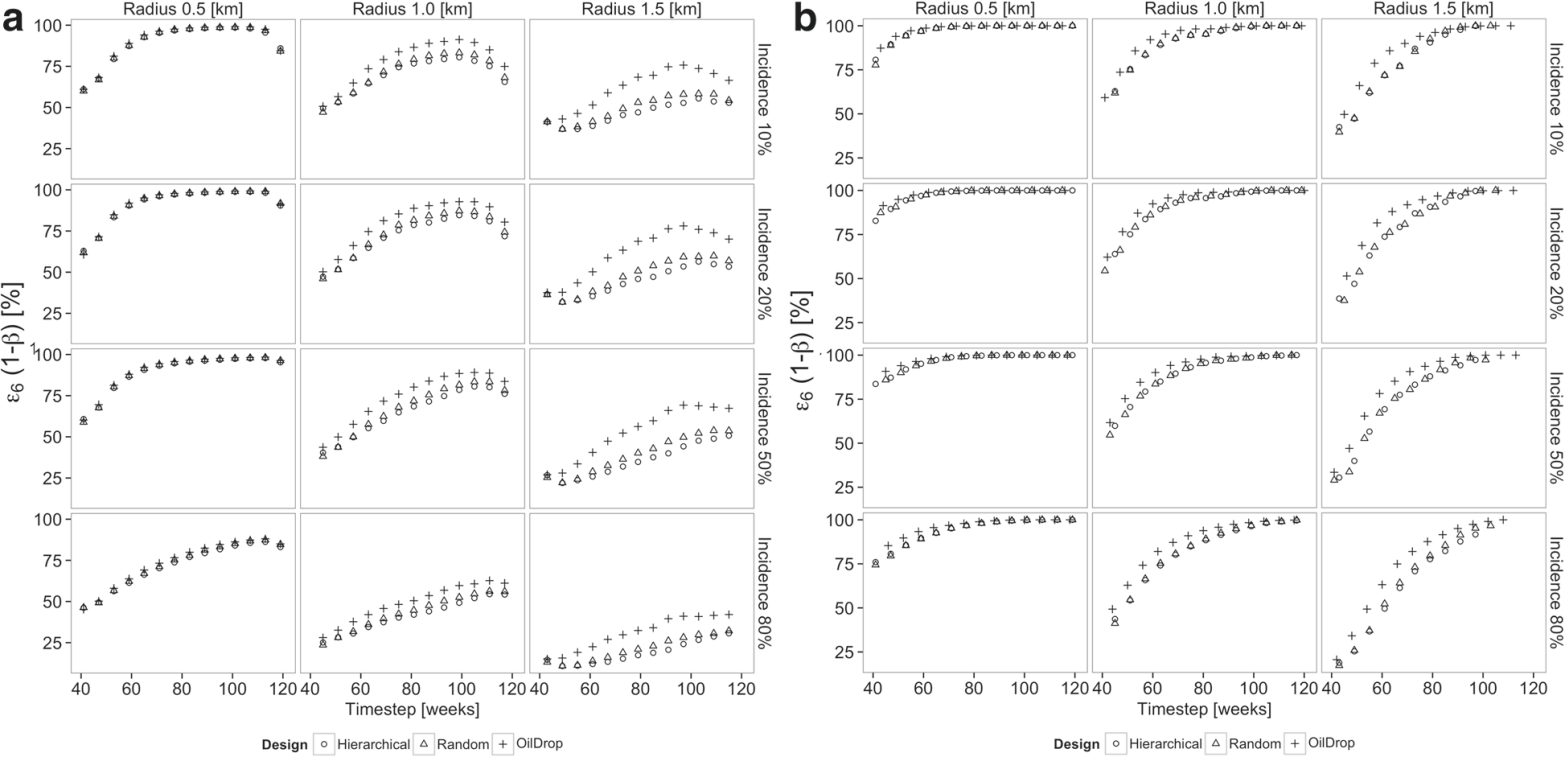
Power over time of contemporaneous effectiveness measure *ε*_6_(*t*) to detect a difference between the naive individuals close to the intervention and those remote from the intervention. All simulations are based on a cluster diameter of 1 km and one-sided type I error rate *α* = 5 %. Open circle: hierarchical ordering; plus sign: oil-drop ordering; open triangle: random ordering. **a** Intervention efficacy of 30 %; **b** intervention efficacy of 80 %

The power of both $\hat {e}_{5}(t)$ and $\hat {e}_{6}(t)$ both increase throughout the rollout in most of the settings shown in Figs. 5 and 6, though in some cases there is a loss of power towards the end, when the comparator groups become small. The primary drivers of the power of a measure are thus the efficacy, the initial incidence, and community effect radius, regardless of design, with results becoming less consistent at community radii of greater than half the cluster diameter.

### Results of simulations for the SolarMal trial

All three study design structures were simulated across the Rusinga landscape, with similar relationships seen among the designs simulated across the theoretical grid. The overall evaluation with the project team of both operational and statistical considerations led us to conclude that the best design for the SolarMal project would be the hierarchical SWCRT. The logistics of the SolarMal project were such that one meta-cluster, comprising nine clusters, could be completed on average every three months

**Fig. 7:**
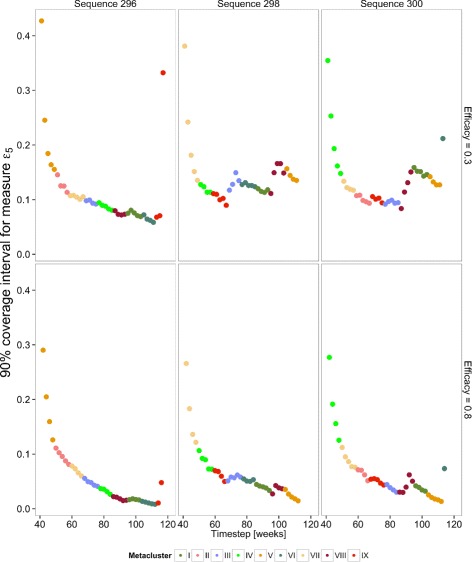
Sequence selection. Three hierarchical sequences applied across Rusinga Island for two levels of intervention efficacy, 30 % and 80 %. Results are color coded by the meta-cluster membership. Reading from the left, the meta-cluster sequences are [V, II, VII, III, IV, VIII, I, VI, IX], [V, IV, IX, III, VI, I, VIII, V, II], and [IV, VII, II, IX, III, VIII, I, V, VI]. Coverage intervals widen upon introduction of the intervention to meta-cluster VIII, located at the base of the peninsula in the north-east of the island, in the right two sequences. Coverage intervals were off the scale for the last meta-cluster of sequence 298. Of the three cluster sequences presented, only sequence 296 met the criteria for entry into the pool for the SolarMal randomization sequence selection lottery (coverage intervals of the primary effectiveness measure, *ε*_5_, less than 10 % from time points 60 to 100, and no single village rollout greater than 6 months’ duration)

Hierarchical sequences were ranked inversely on the basis of the maximum confidence interval width for $ \hat {e}_{5}(t) $ between simulation time steps 60 and 100. Approximately 1/3 of the hierarchical sequences examined met this minimal criterion; a further sociological constraint, that members within a single village receive the intervention within a 6-month time frame was considerably more restrictive. From a total of 10,000 sequences evaluated, 55 met this requirement. Furthermore, each meta-cluster was to have equal chances of being selected first for the intervention lottery; this restraint reduced the final set of acceptable sequences to 27. Examination of the confidence interval width versus time graphs showed wide variation among sequences that can be directly related to the geography of Rusinga. Certain geographic features reappear consistently in the effectiveness graphs as the design is rolled out. For example, when meta-cluster VIII, located at the base of the peninsula in the north-east corner of the island, appears in the last half of a randomization sequence, the precision of the estimated effectiveness rapidly decreases; see Fig. 7. One should expect similar geographic signatures to be found in future geographically informed trial designs.

## Discussion

CRTs are widely used to evaluate interventions against infectious agents (such as hygiene or vector control measures) because of well-known ethical and logistic limitations of individually randomized controlled trials in evaluating health interventions that are applied at the level of the population or group [3]. The present study proposes two extensions to the usual CRT design.

Firstly, we propose that the collection of outcome data should include zones where contamination is likely to occur. Contamination between intervention and control arms is generally seen as something to avoid in CRTs, leading to attempts to separate the study arms with buffer zones [17]. However, CRTs with buffer zones provide information only about the effects of a fixed level of coverage and of a single cluster geometry. It can be difficult to exclude the possibility that contamination substantially biases estimates of effect, since this bias, in the general case, cannot be estimated from the trial data. Rather than struggling to avoid the impact of such unknown community effects, we propose that explicit measurement of the treatment effectiveness in the boundary zones between intervention and control areas should be used to estimate these effects in space and time. Zones of imperfect coverage are needed if inferences are to be made about the radius of effect, the relative magnitudes of individual and community level effects, or the temporal dynamics of spillover effects.

Secondly, we note that local elimination of a pathogen is a single all-or-nothing outcome at the level of a whole area, so an empirical refutation of its feasibility requires scale-up to universal coverage and cannot be achieved if there are untreated control clusters. If elimination proves not to be achievable, it is important to be able to estimate how near the attempt was to success. Conversely, elimination may be achievable at some coverage less than the maximum that can be reached, in which case there is a need to identify this coverage, and to understand what would be needed elsewhere. This specific requirement to consider the impact of maximal coverage over a wide area provides a strong rationale for adopting SWCRT designs for addressing the feasibility of pathogen elimination, additional to the questions of power, bias, and efficiency usually considered in the debate between proponents of parallel designs and of SWCRTs [18–21].

It is often difficult to gain acceptance for CRTs in operational settings because program managers generally aim for complete coverage [21] and hence, tend to evaluate programs using simple before-and-after designs. SWCRT designs are under-exploited because program implementers often do not appreciate the importance of randomization, which is critical for inferring causality. They have more immediate concerns in getting programs off the ground, and only appreciate the need for inference about the effects of the intervention after the event [22]. The SolarMal trial is one situation where this is not the case, and provided an opportunity to implement a widespread intervention trial with careful attention to design.

The evaluation of the distances over which community effects operate in the ITN trials [7–9] provides a basis for evaluating the sizes for estimated community radii for the SolarMal trial, since we assume that community effects of OBTs and of ITNs result from the same phenomena of mosquito dispersion while foraging for food (nectar and blood) and oviposition sites. In a larger malaria control trial in Asembo, close to the SolarMal site, effects were found for distances up to 900 m from cluster boundaries [8], while on the Kenyan coast significant effects persisted for distances up to 1.5 km [9]. Our simulations suggest the precision of the effectiveness measures is robust to variations in community radius above 1 km and that clusters with radii greater than 1 km should be used in such trials. Rusinga Island is, however, large enough for only about nine clusters of this size, and nine clusters would not provide a sufficient degree of replication for a standard CRT.

The use of a stepped wedge means that much smaller individual clusters can be used than in a conventional parallel design of CRT, since as the intervention is rolled out, adjoining clusters are assigned to the intervention, and the radius of intervened areas grows. This also motivated us to consider the oil-drop design, in which the intervention spreads out across the whole area from a single randomly chosen point. While this approach is unbiased over repeated sampling, correlation between the geographical pattern in disease incidence and the rollout pattern is likely to make such a design difficult to interpret. Conversely, for the SolarMal trial, a completely random order of assignment of the 81 clusters would have led to intervened areas that are too fragmented for much of the period of scale-up (and also violated the community’s desire to limit asynchronicity of introduction within a village). The hierarchical SWCRT with nine meta-clusters each divided into nine clusters, represents a compromise that may increase the information obtainable from analyses of the spatial effects of the OBTs across cluster boundaries, while reducing the risk of a strong correlation between baseline disease incidence and rollout pattern.

Further analysis is needed to determine how to optimize such designs given this trade-off between the benefits of independent allocation of clusters and optimal geometry of the intervened areas. It is not obvious how to assess the implications for causal inference of the dependent assignment of clusters in the hierarchical and oil-drop designs. Since the geometry of the intervened areas is time-dependent, the seasonality of the disease is also relevant, and although our limited analysis did not find substantial effects of spatial heterogeneity in population density or disease transmission on the precision of the effectiveness measures, these remain factors that should be considered. For the SolarMal study, we did not aspire to achieve optimality and a number of possible designs and sequences were simulated. Various metrics of the power of each effectiveness measure to estimate the spatial effect of the intervention on clinical malaria incidence were assessed, and a set of the preferred sequences of the hierarchical design was presented to community representatives as alternatives, and the one to be implemented was drawn by lot.

The contemporaneous comparison of intervened versus naive areas ($\hat {e}_{4}(t)$) should, in general, be considered the primary outcome of a SWCRT, but the quantification of effects at different levels of proximity to the intervention and over time using $\hat {e}_{6}(t)$ is likely to prove invaluable for parameterizing models of the effects of varying coverage in space and time. An extension of such empirical time and space models will be to include time-weighted lags in the effects of coverage, akin to the modeling of SWCRT proposed by Hussey and Hughes [3]. This will allow generalized prediction of the likely impact of different patterns of coverage of OBTs in space and time. The broad principles of the analysis will be similar for different outcomes: densities of host-seeking mosquitoes (as measured by sentinel OBTs), parasite positivity (by a rapid diagnostic test), malaria fever incidence, and all-cause mortality. Another extension of this work would be to develop analytical formulae for interval estimation of the novel outcome measures, and to assess their nominal coverage against intervals obtained (as in this paper) from repeated simulations. However, in practice, the model-based confidence intervals described above, or sampling-based approaches such as bootstrapping or Bayesian Markov chain Monte Carlo, provide alternatives to the development of such bespoke methods. Sampling-based approaches are especially attractive since they can easily be applied to extended models incorporating lags and covariates. A program in R that can be used to simulate trials with different values of $\overline {y}(0)$, *E*_*s*_, *r*, and *N*(*T*) is provided as Additional file 2. This program could be adapted both to consider further effects of spatial and temporal heterogeneity in risk, and also for the design of other trials with different geographies.

## Conclusions

Contamination between arms in CRTs can be a source of information about the effects of incomplete coverage, and can provide supporting evidence for causal inference. It follows that trials should be designed with such analyses in mind, and contamination should not be seen simply as a problem to be avoided. Where scale-up to complete coverage is required, as in assessments of the feasibility of local elimination of a pathogen, the SWCRT is an appropriate design. This leads to temporal changes in which zones are affected by contamination. The SolarMal example illustrates how generic transmission models incorporating spatial smoothing can be used to simulate such trials for a power calculation and optimization of cluster size and randomization strategies. The approach is applicable to a range of infectious diseases transmitted via environmental reservoirs or via arthropod vectors.

## Additional files

Additional file 1 Supplementary tables and figures. Results of additional analyses referred to in the text. (PDF 132 kb)

Additional file 2 Documented R code for simulating trials analogous to the example given in the text. (R 676 kb)

## Abbreviations

CRT: cluster randomized trial
ITN: insecticide-treated net
OBT: odor-baited trap
SolarMal: Solar Power for Malaria Control trial
SWCRT: stepped wedge cluster randomized trial
