# Supplementary material for: Design of trials for interrupting the transmission of endemic pathogens
Source: Trials. 2016 Jun 6;17:278. doi: 10.1186/s13063-016-1378-1 (PMC4895826; doi:10.1186/s13063-016-1378-1)
Supplement: Additional file 1 — Supplementary tables and figures. Results of additional analyses referred to in the text. (PDF 132 kb) [file 13063_2016_1378_MOESM1_ESM.pdf]

Tables

Table S1: Power at rollout week 60 (simulation week 100) for the hierarchical design

Simulated effectiveness: $\hat{e}_i = 30\%$, significance level $\alpha = 0.1$							
r	$\bar{y_r}(0)$	$\hat{e}_1$	$\hat{e}_2$	$\hat{e}_3$	$\hat{e}_4$	$\hat{e}_5$	$\hat{e}_6$
0.50	0.10	1.00	0.94	1.00	0.98	0.89	0.52
0.50	0.20	1.00	0.93	1.00	0.99	0.90	0.52
0.50	0.50	0.99	0.80	1.00	0.97	0.83	0.40
0.50	0.80	0.95	0.41	0.99	0.84	0.58	0.18
1.00	0.10	1.00	0.99	1.00	0.80	0.80	0.66
1.00	0.20	1.00	0.98	1.00	0.84	0.73	0.58
1.00	0.50	0.99	0.95	1.00	0.79	0.63	0.45
1.00	0.80	0.91	0.66	0.99	0.50	0.44	0.29
1.50	0.10	1.00	0.99	1.00	0.54	0.94	0.88
1.50	0.20	0.99	0.99	1.00	0.54	0.79	0.74
1.50	0.50	0.98	0.97	1.00	0.45	0.56	0.48
1.50	0.80	0.88	0.78	0.99	0.25	0.41	0.36
Simulated effectiveness: $\hat{e}_i = 80\%$,$\alpha = 0.1$							
r	$\bar{y_r}(0)$	$\hat{e}_1$	$\hat{e}_2$	$\hat{e}_3$	$\hat{e}_4$	$\hat{e}_5$	$\hat{e}_6$
0.50	0.10	1.00	0.99	1.00	1.00	1.00	0.82
0.50	0.20	1.00	0.99	1.00	1.00	1.00	0.84
0.50	0.50	1.00	0.98	1.00	1.00	1.00	0.81
0.50	0.80	1.00	0.91	1.00	1.00	1.00	0.67
1.00	0.10	1.00	1.00	1.00	0.99	1.00	0.92
1.00	0.20	1.00	1.00	1.00	1.00	1.00	0.87
1.00	0.50	1.00	1.00	1.00	1.00	0.99	0.80
1.00	0.80	1.00	0.99	1.00	1.00	0.97	0.72
1.50	0.10	1.00	1.00	1.00	0.86	1.00	1.00
1.50	0.20	1.00	1.00	1.00	0.93	1.00	0.99
1.50	0.50	1.00	1.00	1.00	0.97	0.99	0.87
1.50	0.80	1.00	0.99	1.00	0.97	0.94	0.77

Table S2: Power at rollout week 60 (simulation week 100) for the oil drop design

Simulated effectiveness: $\hat{e}_i = 30\%, \alpha = 0.1$							
r	$\bar{y_r}(0)$	$\hat{e}_1$	$\hat{e}_2$	$\hat{e}_3$	$\hat{e}_4$	$\hat{e}_5$	$\hat{e}_6$
0.50	0.10	1.00	0.90	1.00	0.99	0.93	0.53
0.50	0.20	1.00	0.89	1.00	0.99	0.94	0.54
0.50	0.50	0.99	0.72	1.00	0.98	0.88	0.41
0.50	0.80	0.95	0.37	1.00	0.86	0.64	0.18
1.00	0.10	1.00	0.97	1.00	0.91	0.84	0.64
1.00	0.20	1.00	0.97	1.00	0.93	0.82	0.59
1.00	0.50	0.99	0.92	1.00	0.88	0.74	0.48
1.00	0.80	0.93	0.60	0.99	0.60	0.52	0.28
1.50	0.10	1.00	0.98	1.00	0.75	0.84	0.63
1.50	0.20	1.00	0.98	1.00	0.77	0.82	0.61
1.50	0.50	0.99	0.95	1.00	0.69	0.76	0.51
1.50	0.80	0.91	0.71	0.99	0.41	0.52	0.31
Simulated effectiveness: $\hat{e}_i = 80\%, \alpha = 0.1$							
r	$\bar{y_r}(0)$	$\hat{e}_1$	$\hat{e}_2$	$\hat{e}_3$	$\hat{e}_4$	$\hat{e}_5$	$\hat{e}_6$
0.50	0.10	1.00	0.98	1.00	1.00	1.00	0.84
0.50	0.20	1.00	0.98	1.00	1.00	1.00	0.87
0.50	0.50	1.00	0.97	1.00	1.00	1.00	0.84
0.50	0.80	1.00	0.86	1.00	1.00	1.00	0.68
1.00	0.10	1.00	1.00	1.00	0.99	1.00	0.88
1.00	0.20	1.00	1.00	1.00	1.00	1.00	0.87
1.00	0.50	1.00	0.99	1.00	1.00	0.99	0.84
1.00	0.80	1.00	0.97	1.00	1.00	0.99	0.75
1.50	0.10	1.00	1.00	1.00	0.94	1.00	0.88
1.50	0.20	1.00	1.00	1.00	0.97	1.00	0.88
1.50	0.50	1.00	1.00	1.00	0.99	1.00	0.90
1.50	0.80	1.00	0.99	1.00	0.98	0.99	0.82

Table S3: Power at rollout week 60 (simulation week 100) for the random design

Simulated effectiveness: $\hat{e}_i = 30\%, \alpha = 0.1$							
r	$\bar{y_r}(0)$	$\hat{e}_1$	$\hat{e}_2$	$\hat{e}_3$	$\hat{e}_4$	$\hat{e}_5$	$\hat{e}_6$
0.50	0.10	1.00	0.93	1.00	0.99	0.90	0.51
0.50	0.20	1.00	0.93	1.00	0.99	0.90	0.52
0.50	0.50	0.99	0.79	1.00	0.98	0.83	0.41
0.50	0.80	0.95	0.41	1.00	0.85	0.59	0.18
1.00	0.10	1.00	0.99	1.00	0.83	0.82	0.67
1.00	0.20	1.00	0.98	1.00	0.87	0.74	0.56
1.00	0.50	0.99	0.95	1.00	0.81	0.64	0.45
1.00	0.80	0.92	0.66	0.99	0.53	0.45	0.29
1.50	0.10	1.00	0.99	1.00	0.58	0.95	0.91
1.50	0.20	1.00	0.99	1.00	0.59	0.86	0.78
1.50	0.50	0.99	0.97	1.00	0.50	0.62	0.51
1.50	0.80	0.90	0.78	0.99	0.28	0.45	0.37
Simulated effectiveness: $\hat{e}_i = 80\%, \alpha = 0.1$							
r	$\bar{y_r}(0)$	$\hat{e}_1$	$\hat{e}_2$	$\hat{e}_3$	$\hat{e}_4$	$\hat{e}_5$	$\hat{e}_6$
0.50	0.10	1.00	0.99	1.00	1.00	1.00	0.82
0.50	0.20	1.00	0.99	1.00	1.00	1.00	0.84
0.50	0.50	1.00	0.98	1.00	1.00	1.00	0.81
0.50	0.80	1.00	0.91	1.00	1.00	1.00	0.67
1.00	0.10	1.00	1.00	1.00	0.99	1.00	0.92
1.00	0.20	1.00	1.00	1.00	1.00	1.00	0.87
1.00	0.50	1.00	1.00	1.00	1.00	0.99	0.80
1.00	0.80	1.00	0.99	1.00	1.00	0.97	0.72
1.50	0.10	1.00	1.00	1.00	0.86	1.00	1.00
1.50	0.20	1.00	1.00	1.00	0.93	1.00	0.99
1.50	0.50	1.00	1.00	1.00	0.97	0.99	0.87
1.50	0.80	1.00	0.99	1.00	0.97	0.94	0.77

Table S4: Median effectiveness measures and 95% confidence interval widths for the hierarchical design, summarized over 80 replicates and 10 geographies

r	E_s	$\bar{y_r}(0)$	$\hat{e}_1$	$\hat{e}_2$	$\hat{e}_3$	$\hat{e}_1$ 95% CI width	$\hat{e}_2$ 95% CI width	$\hat{e}_3$ 95% CI width	$\hat{e}_4$	$\hat{e}_5$	$\hat{e}_6$	$\hat{e}_4$ CI width	$\hat{e}_5$ CI width	$\hat{e}_6$ CI width
0.5	0.3	0.1	0.35	0.08	0.14	0.31	0.23	0.20	0.30	0.32	0.12	0.36	0.38	0.43
0.5	0.3	0.2	0.33	0.06	0.13	0.21	0.15	0.13	0.28	0.31	0.11	0.24	0.25	0.28
0.5	0.3	0.5	0.25	0.03	0.09	0.12	0.07	0.07	0.22	0.24	0.08	0.13	0.13	0.14
0.5	0.3	0.8	0.15	0.02	0.05	0.07	0.03	0.04	0.13	0.15	0.04	0.07	0.07	0.07
0.5	0.8	0.1	0.74	0.14	0.28	0.20	0.23	0.21	0.70	0.72	0.29	0.23	0.22	0.37
0.5	0.8	0.2	0.72	0.12	0.27	0.15	0.15	0.15	0.68	0.71	0.28	0.17	0.16	0.26
0.5	0.8	0.5	0.66	0.08	0.23	0.12	0.08	0.11	0.63	0.66	0.22	0.13	0.12	0.14
0.5	0.8	0.8	0.55	0.05	0.17	0.11	0.04	0.09	0.53	0.55	0.14	0.11	0.11	0.08
1.0	0.3	0.1	0.23	0.09	0.12	0.32	0.23	0.21	0.16	0.21	0.10	0.37	0.46	0.44
1.0	0.3	0.2	0.21	0.08	0.11	0.22	0.15	0.13	0.14	0.20	0.09	0.25	0.31	0.29
1.0	0.3	0.5	0.16	0.05	0.08	0.12	0.07	0.07	0.11	0.16	0.07	0.14	0.16	0.14
1.0	0.3	0.8	0.09	0.03	0.04	0.07	0.03	0.03	0.06	0.09	0.04	0.07	0.09	0.07
1.0	0.8	0.1	0.54	0.21	0.29	0.26	0.23	0.21	0.42	0.52	0.25	0.35	0.34	0.39
1.0	0.8	0.2	0.52	0.19	0.27	0.20	0.16	0.16	0.40	0.50	0.23	0.26	0.25	0.26
1.0	0.8	0.5	0.44	0.15	0.22	0.15	0.10	0.12	0.34	0.43	0.19	0.18	0.17	0.14
1.0	0.8	0.8	0.31	0.09	0.14	0.12	0.06	0.08	0.24	0.31	0.11	0.13	0.13	0.08
1.5	0.3	0.1	0.17	0.10	0.12	0.33	0.23	0.21	0.07	0.15	0.09	0.39	0.78	0.77
1.5	0.3	0.2	0.16	0.09	0.11	0.22	0.15	0.14	0.07	0.14	0.09	0.26	0.51	0.50
1.5	0.3	0.5	0.12	0.07	0.08	0.12	0.07	0.07	0.05	0.11	0.07	0.14	0.25	0.24
1.5	0.3	0.8	0.06	0.03	0.04	0.06	0.04	0.03	0.03	0.06	0.04	0.07	0.13	0.12
1.5	0.8	0.1	0.41	0.25	0.29	0.30	0.23	0.22	0.22	0.39	0.24	0.42	0.61	0.66
1.5	0.8	0.2	0.40	0.24	0.28	0.23	0.17	0.17	0.20	0.37	0.23	0.30	0.42	0.44
1.5	0.8	0.5	0.32	0.18	0.22	0.16	0.11	0.12	0.17	0.31	0.18	0.19	0.25	0.23
1.5	0.8	0.8	0.20	0.11	0.13	0.12	0.07	0.08	0.11	0.20	0.11	0.12	0.16	0.13

Table S5: Bias defined as the difference between median estimates and median $\hat{e}_1$, hierarchical design, summarized over 80 replicates and 10 geographies

r	E_s	$\bar{y_r}(0)$	Bias $\hat{e}_2$	Bias$\hat{e}_3$	Bias $\hat{e}_4$	Bias $\hat{e}_5$	Bias $\hat{e}_6$
0.5	0.3	0.1	0.28	0.21	0.05	0.03	0.24
0.5	0.3	0.2	0.26	0.20	0.05	0.02	0.22
0.5	0.3	0.5	0.22	0.16	0.03	0.01	0.17
0.5	0.3	0.8	0.13	0.10	0.01	0.00	0.10
0.5	0.8	0.1	0.60	0.45	0.04	0.01	0.45
0.5	0.8	0.2	0.60	0.45	0.04	0.01	0.45
0.5	0.8	0.5	0.58	0.44	0.03	0.01	0.44
0.5	0.8	0.8	0.50	0.38	0.02	0.00	0.41
1.0	0.3	0.1	0.14	0.11	0.08	0.02	0.14
1.0	0.3	0.2	0.13	0.10	0.07	0.01	0.12
1.0	0.3	0.5	0.11	0.08	0.05	0.01	0.09
1.0	0.3	0.8	0.06	0.05	0.03	0.00	0.05
1.0	0.8	0.1	0.33	0.25	0.12	0.02	0.29
1.0	0.8	0.2	0.32	0.24	0.12	0.02	0.28
1.0	0.8	0.5	0.29	0.22	0.10	0.01	0.26
1.0	0.8	0.8	0.22	0.17	0.07	0.00	0.20
1.5	0.3	0.1	0.07	0.05	0.10	0.02	0.08
1.5	0.3	0.2	0.06	0.05	0.09	0.02	0.07
1.5	0.3	0.5	0.05	0.04	0.06	0.01	0.05
1.5	0.3	0.8	0.03	0.02	0.03	0.00	0.03
1.5	0.8	0.1	0.16	0.12	0.20	0.03	0.17
1.5	0.8	0.2	0.16	0.12	0.19	0.03	0.17
1.5	0.8	0.5	0.14	0.10	0.15	0.01	0.13
1.5	0.8	0.8	0.09	0.07	0.10	0.00	0.09

Table S6: Median effectiveness measures and 95% confidence interval widths for the oil drop design, summarized over 80 replicates and 10 geographies

r	E_s	$\bar{y_r}(0)$ Prevalence	$\hat{e}_1$	$\hat{e}_2$	$\hat{e}_3$	$\hat{e}_1$ CI width	$\hat{e}_2$ CI width	$\hat{e}_3$ CI width	$\hat{e}_4$	$\hat{e}_5$	$\hat{e}_6$	$\hat{e}_4$ CI width	$\hat{e}_5$ CI width	$\hat{e}_6$ CI width
0.5	0.3	0.1	0.36	0.07	0.14	0.31	0.23	0.20	0.31	0.34	0.12	0.36	0.37	0.44
0.5	0.3	0.2	0.34	0.06	0.13	0.21	0.15	0.13	0.30	0.32	0.11	0.25	0.25	0.29
0.5	0.3	0.5	0.26	0.03	0.09	0.12	0.07	0.06	0.24	0.25	0.08	0.14	0.13	0.14
0.5	0.3	0.8	0.15	0.01	0.05	0.07	0.03	0.03	0.14	0.15	0.04	0.08	0.08	0.07
0.5	0.8	0.1	0.76	0.12	0.28	0.19	0.23	0.19	0.72	0.74	0.29	0.23	0.21	0.39
0.5	0.8	0.2	0.74	0.11	0.27	0.15	0.15	0.13	0.71	0.73	0.28	0.18	0.17	0.27
0.5	0.8	0.5	0.68	0.08	0.23	0.12	0.08	0.08	0.66	0.68	0.23	0.14	0.13	0.15
0.5	0.8	0.8	0.57	0.04	0.17	0.13	0.04	0.05	0.55	0.57	0.14	0.14	0.13	0.09
1.0	0.3	0.1	0.25	0.08	0.12	0.32	0.24	0.20	0.19	0.24	0.10	0.39	0.45	0.41
1.0	0.3	0.2	0.24	0.07	0.11	0.23	0.15	0.13	0.18	0.23	0.09	0.27	0.29	0.27
1.0	0.3	0.5	0.18	0.05	0.08	0.13	0.07	0.06	0.14	0.18	0.07	0.15	0.16	0.13
1.0	0.3	0.8	0.10	0.03	0.04	0.07	0.03	0.03	0.08	0.10	0.04	0.09	0.09	0.07
1.0	0.8	0.1	0.58	0.19	0.28	0.27	0.23	0.19	0.49	0.57	0.26	0.38	0.33	0.37
1.0	0.8	0.2	0.56	0.17	0.27	0.23	0.16	0.13	0.47	0.55	0.25	0.31	0.26	0.26
1.0	0.8	0.5	0.49	0.13	0.22	0.20	0.09	0.08	0.40	0.48	0.20	0.26	0.22	0.14
1.0	0.8	0.8	0.35	0.08	0.15	0.19	0.05	0.05	0.29	0.35	0.12	0.23	0.20	0.08
1.5	0.3	0.1	0.20	0.09	0.12	0.33	0.22	0.20	0.12	0.19	0.09	0.40	0.58	0.54
1.5	0.3	0.2	0.18	0.09	0.11	0.23	0.14	0.13	0.11	0.18	0.09	0.28	0.39	0.36
1.5	0.3	0.5	0.14	0.06	0.08	0.13	0.07	0.06	0.08	0.14	0.07	0.16	0.20	0.17
1.5	0.3	0.8	0.08	0.03	0.04	0.08	0.03	0.03	0.04	0.08	0.04	0.09	0.11	0.09
1.5	0.8	0.1	0.47	0.23	0.29	0.32	0.23	0.19	0.32	0.47	0.26	0.47	0.48	0.47
1.5	0.8	0.2	0.45	0.22	0.27	0.26	0.17	0.13	0.30	0.44	0.25	0.39	0.36	0.33
1.5	0.8	0.5	0.37	0.17	0.22	0.22	0.10	0.08	0.24	0.37	0.20	0.32	0.26	0.18
1.5	0.8	0.8	0.25	0.10	0.14	0.20	0.06	0.05	0.16	0.24	0.12	0.25	0.22	0.10

Table S7: Bias defined as the difference between median effectiveness estimates and median $\hat{e}_1$, oil drop design, summarized over 80 replicates and 10 geographies

r	E_s	$\bar{y}_r(0)$	Bias $\hat{e}_2$	Bias $\hat{e}_3$	Bias $\hat{e}_4$	Bias $\hat{e}_5$	Bias $\hat{e}_6$
0.5	0.3	0.1	0.29	0.22	0.05	0.03	0.25
0.5	0.3	0.2	0.28	0.21	0.04	0.02	0.23
0.5	0.3	0.5	0.23	0.17	0.02	0.01	0.18
0.5	0.3	0.8	0.14	0.10	0.01	0.00	0.11
0.5	0.8	0.1	0.63	0.47	0.04	0.01	0.46
0.5	0.8	0.2	0.63	0.48	0.03	0.01	0.46
0.5	0.8	0.5	0.61	0.46	0.03	0.00	0.46
0.5	0.8	0.8	0.53	0.40	0.02	0.00	0.43
1.0	0.3	0.1	0.17	0.13	0.07	0.01	0.15
1.0	0.3	0.2	0.16	0.12	0.06	0.01	0.14
1.0	0.3	0.5	0.13	0.10	0.04	0.00	0.11
1.0	0.3	0.8	0.08	0.06	0.02	0.00	0.06
1.0	0.8	0.1	0.40	0.30	0.10	0.01	0.32
1.0	0.8	0.2	0.39	0.29	0.09	0.01	0.31
1.0	0.8	0.5	0.35	0.26	0.08	0.01	0.29
1.0	0.8	0.8	0.27	0.20	0.06	0.00	0.23
1.5	0.3	0.1	0.11	0.08	0.08	0.01	0.11
1.5	0.3	0.2	0.10	0.07	0.08	0.01	0.09
1.5	0.3	0.5	0.08	0.06	0.06	0.00	0.07
1.5	0.3	0.8	0.04	0.03	0.03	0.00	0.04
1.5	0.8	0.1	0.24	0.18	0.16	0.01	0.21
1.5	0.8	0.2	0.24	0.18	0.15	0.01	0.21
1.5	0.8	0.5	0.21	0.15	0.13	0.00	0.18
1.5	0.8	0.8	0.15	0.11	0.09	0.00	0.13

Table S8: Median effectiveness measures and 95% confidence interval widths for the random design, summarized over 80 replicates and 10 geographies

r	E_s	$\bar{y}_r(0)$	$\hat{e}_1$	$\hat{e}_2$	$\hat{e}_3$	$\hat{e}_1$ CI width	$\hat{e}_2$ CI width	$\hat{e}_3$ CI width	$\hat{e}_4$	$\hat{e}_5$	$\hat{e}_6$	$\hat{e}_4$ CI width	$\hat{e}_5$ CI width	$\hat{e}_6$ CI width
0.5	0.3	0.1	0.35	0.08	0.14	0.30	0.23	0.20	0.30	0.32	0.12	0.36	0.37	0.43
0.5	0.3	0.2	0.33	0.06	0.13	0.21	0.15	0.13	0.28	0.31	0.11	0.25	0.25	0.27
0.5	0.3	0.5	0.25	0.04	0.09	0.11	0.07	0.06	0.22	0.24	0.08	0.13	0.13	0.14
0.5	0.3	0.8	0.15	0.02	0.05	0.07	0.03	0.03	0.13	0.15	0.04	0.07	0.07	0.07
0.5	0.8	0.1	0.74	0.14	0.28	0.19	0.23	0.19	0.70	0.73	0.29	0.23	0.21	0.37
0.5	0.8	0.2	0.72	0.12	0.27	0.15	0.15	0.13	0.69	0.71	0.27	0.17	0.16	0.25
0.5	0.8	0.5	0.66	0.08	0.23	0.11	0.07	0.07	0.63	0.66	0.22	0.13	0.12	0.14
0.5	0.8	0.8	0.55	0.05	0.17	0.11	0.04	0.05	0.53	0.55	0.14	0.12	0.11	0.08
1.0	0.3	0.1	0.24	0.09	0.12	0.32	0.23	0.20	0.16	0.22	0.10	0.37	0.46	0.44
1.0	0.3	0.2	0.22	0.08	0.11	0.22	0.14	0.13	0.15	0.20	0.09	0.26	0.31	0.28
1.0	0.3	0.5	0.16	0.06	0.08	0.12	0.07	0.06	0.11	0.16	0.07	0.14	0.16	0.14
1.0	0.3	0.8	0.09	0.03	0.04	0.07	0.03	0.03	0.07	0.09	0.04	0.08	0.09	0.07
1.0	0.8	0.1	0.54	0.21	0.29	0.26	0.22	0.18	0.42	0.53	0.25	0.36	0.34	0.38
1.0	0.8	0.2	0.52	0.19	0.28	0.20	0.15	0.13	0.41	0.51	0.23	0.28	0.25	0.26
1.0	0.8	0.5	0.45	0.15	0.22	0.16	0.08	0.08	0.35	0.44	0.19	0.20	0.18	0.14
1.0	0.8	0.8	0.31	0.09	0.14	0.14	0.04	0.05	0.25	0.31	0.11	0.16	0.15	0.08
1.5	0.3	0.1	0.18	0.10	0.12	0.32	0.22	0.20	0.08	0.15	0.09	0.38	0.76	0.76
1.5	0.3	0.2	0.16	0.10	0.11	0.22	0.14	0.13	0.07	0.15	0.09	0.26	0.50	0.49
1.5	0.3	0.5	0.12	0.07	0.08	0.12	0.07	0.06	0.05	0.11	0.07	0.14	0.24	0.22
1.5	0.3	0.8	0.06	0.03	0.04	0.06	0.03	0.03	0.03	0.06	0.04	0.08	0.12	0.11
1.5	0.8	0.1	0.42	0.26	0.30	0.29	0.21	0.19	0.22	0.40	0.24	0.44	0.60	0.64
1.5	0.8	0.2	0.40	0.24	0.28	0.23	0.15	0.13	0.21	0.38	0.23	0.32	0.42	0.42
1.5	0.8	0.5	0.32	0.18	0.22	0.17	0.08	0.08	0.17	0.31	0.18	0.22	0.24	0.21
1.5	0.8	0.8	0.21	0.11	0.13	0.13	0.05	0.05	0.11	0.20	0.11	0.16	0.17	0.12

Table S9: Bias defined as the difference between median $\hat{e}_i$ and median effectiveness, random design, summarized over 80 replicates and 10 geographies

r	E_s	$\bar{y_r}(0)$	Bias $\hat{e}_2$	Bias $\hat{e}_3$	Bias $\hat{e}_4$	Bias $\hat{e}_5$	Bias $\hat{e}_6$
0.5	0.3	0.1	0.27	0.21	0.05	0.03	0.23
0.5	0.3	0.2	0.26	0.20	0.05	0.02	0.22
0.5	0.3	0.5	0.22	0.16	0.03	0.01	0.17
0.5	0.3	0.8	0.13	0.10	0.01	0.00	0.11
0.5	0.8	0.1	0.60	0.45	0.04	0.01	0.45
0.5	0.8	0.2	0.60	0.45	0.04	0.01	0.45
0.5	0.8	0.5	0.58	0.44	0.03	0.01	0.44
0.5	0.8	0.8	0.50	0.38	0.02	0.00	0.41
1.0	0.3	0.1	0.15	0.11	0.08	0.02	0.14
1.0	0.3	0.2	0.14	0.10	0.07	0.01	0.13
1.0	0.3	0.5	0.11	0.08	0.05	0.01	0.10
1.0	0.3	0.8	0.06	0.05	0.03	0.00	0.06
1.0	0.8	0.1	0.34	0.25	0.12	0.02	0.30
1.0	0.8	0.2	0.33	0.25	0.12	0.02	0.29
1.0	0.8	0.5	0.30	0.22	0.10	0.01	0.26
1.0	0.8	0.8	0.23	0.17	0.07	0.00	0.20
1.5	0.3	0.1	0.07	0.05	0.10	0.02	0.08
1.5	0.3	0.2	0.07	0.05	0.09	0.01	0.07
1.5	0.3	0.5	0.05	0.04	0.06	0.01	0.05
1.5	0.3	0.8	0.03	0.02	0.03	0.00	0.03
1.5	0.8	0.1	0.17	0.13	0.20	0.03	0.18
1.5	0.8	0.2	0.16	0.12	0.19	0.02	0.17
1.5	0.8	0.5	0.14	0.11	0.15	0.01	0.14
1.5	0.8	0.8	0.10	0.07	0.10	0.00	0.09

Figures

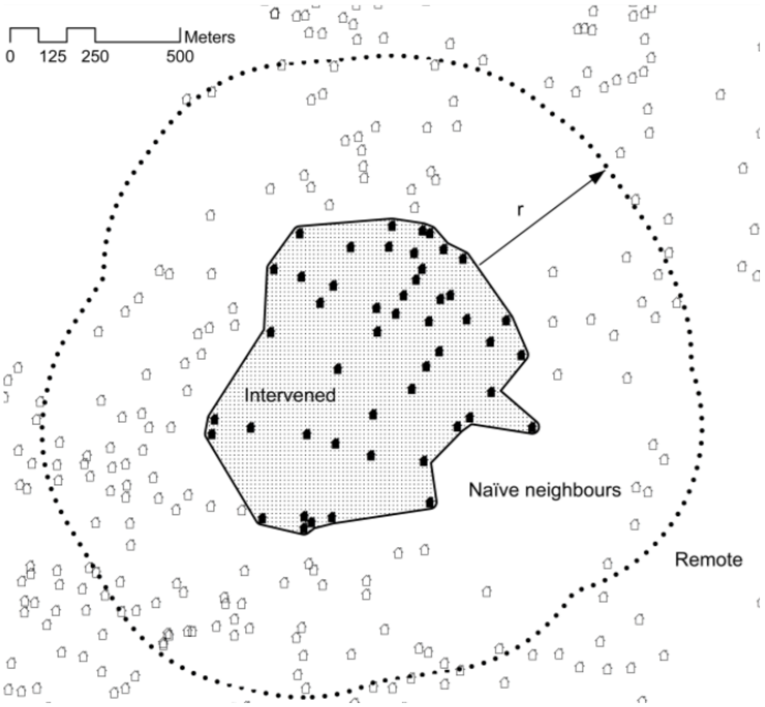

Figure S1: Household assignments during an intermediate time-step of the simulation for a small proportion of the surface illustrating how the intervened, neighbor and remote zones are assigned

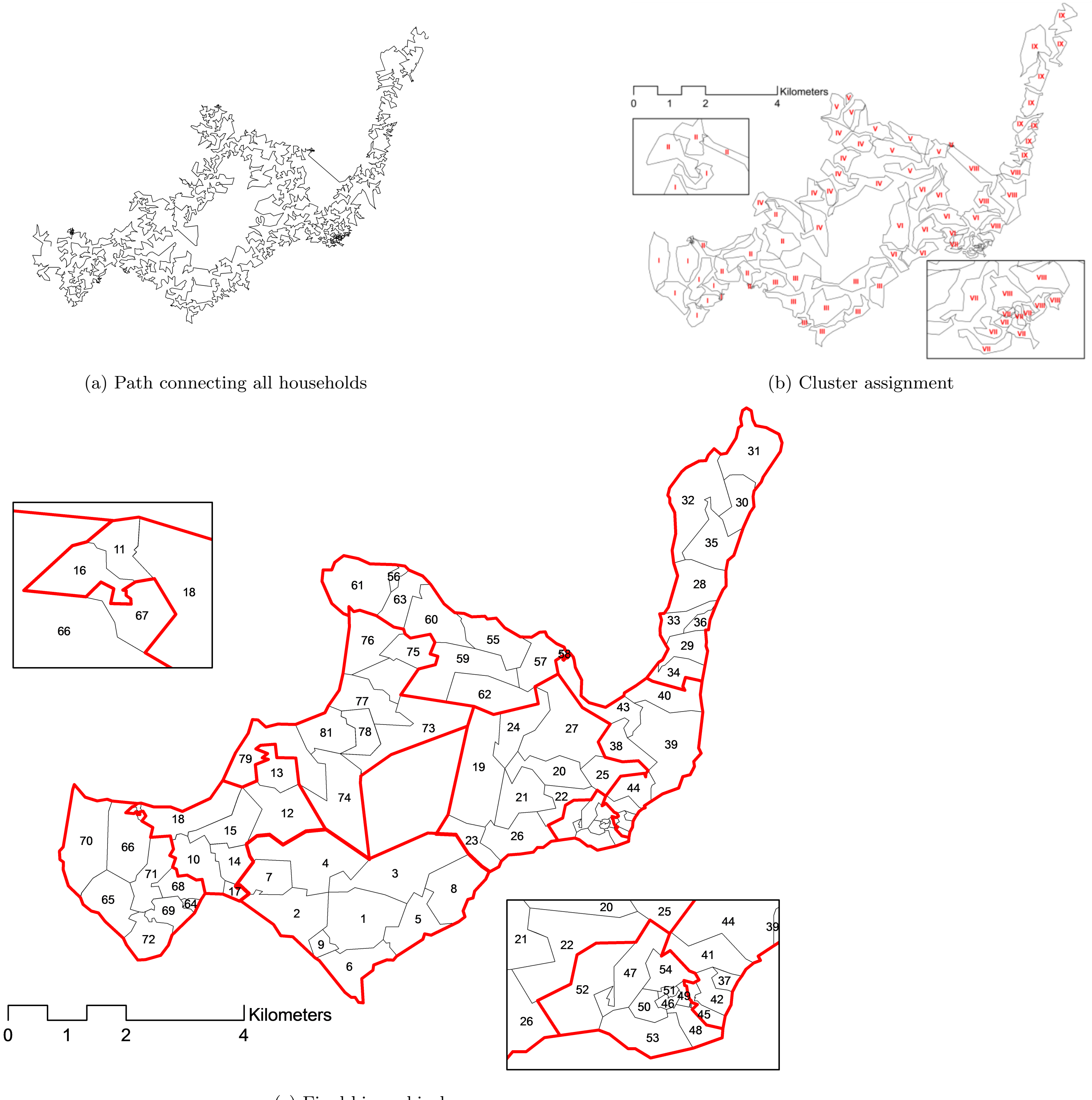

(a) Path connecting all households

(b) Cluster assignment

(c) Final hierarchical sequence

Figure S2: a: minimal spanning tree algorithm applied across the geographic locations of households of Rusinga Island to find an efficient (minimum distance) one-way path connecting all households. b: households were counted off in groups of 50 from a starting point on the south-west corner of the island along the one-way path to construct a total of 81 clusters. Each group of nine clusters is further combined into a metacluster, here denoted from I-IX. c: the final cluster rollout sequence for Rusinga Island is an hierarchical SWCRT. Clusters are identified in the order in which they received the intervention. The unshaded area at graph center is unpopulated.
